# Common allergies in urban adolescents and their relationships with asthma control and healthcare utilization

**DOI:** 10.1186/s13223-018-0260-y

**Published:** 2018-09-03

**Authors:** Hyekyun Rhee, Tanzy Love, Donald Harrington, Annette Grape

**Affiliations:** 1 University of Rochester School of Nursing, 601 Elmwood Ave. Box SON, Rochester, NY 14642 USA; 2 Department of Biostatistics and Computational Biology, University of Rochester Medical Center, 601 Elmwood Ave., Box 630, Rochester, NY 14642 USA

**Keywords:** Urban adolescents, Asthma, Allergies, Cockroach, Mouse, Healthcare utilization

## Abstract

**Background:**

Urban adolescents suffer a disproportionate burden of asthma morbidity, often in association with allergies. Literature is limited on comparing various types of allergies regarding prevalence and associations with asthma morbidity in urban dwelling adolescents. The purpose of this study was to examine the prevalence of common allergies reported by urban adolescents and to assess their relationships to healthcare utilization and asthma control.

**Methods:**

Study participants included 313 urban adolescents (12–20 years of age) with persistent asthma who were recruited from three states in the United States. Self-report data were collected on nine indoor and outdoor allergies, healthcare utilization, and asthma exacerbation. Logistic regressions and zero-inflated Poisson regressions were conducted to examine the relationships between allergies and asthma morbidity.

**Results:**

The mean age of participants was 14.58 (± 1.97) and 52% were female, and 79% were black. Seventy-three percent (n = 229) reported one or more allergies. Dust mite and grass allergies were most common, each reported by 50%. The prevalence of pest allergies (cockroach and mouse) was 27.5% and 19%, respectively. Those with pest allergies were more likely to report ED visits (cockroach- Odds Ratio (OR) = 2.16, 95% CI 1.18–3.94, p = .01; mouse- OR = 2.13, 95% CI 1.09–4.07, p = .02), specialist visits (cockroach-OR = 2.69, 95% CI 1.60–4.54, p < .001; mouse- OR = 2.06, 95% CI 1.15–3.68, p = .01) and asthma exacerbation (cockroach-OR = 2.17, 95% CI 1.26–3.74, p < .001; mouse- OR = 2.30, 95% CI 1.26–4.18, p = .01). Cockroach allergies were associated with 2.2 times as many nights in the hospital (95% CI 1.053–3.398, p = 0.036) and 2.2 times as many specialist visits (95% CI 1.489–3.110, p < 0.001), and mouse allergy was associated with 1.6 times as many ED visits (95% CI 1.092–2.257, p = 0.015) compared to those without pest allergies.

**Conclusions:**

Concomitant occurrence of allergies is ubiquitous among urban adolescents with asthma. Only pest allergies, of those examined, appear to have implications for poorly controlled asthma, exacerbation and acute healthcare utilization. To reduce asthma burden in urban adolescents, identification and management of high-risk adolescents with pest allergen sensitization and exposure are warranted.

## Background

Asthma is a leading chronic pediatric health condition affecting approximately 6.2 million children under age 18 years in the United States (US) [1]. Current asthma is reported in 10% of adolescents (2.5 million) age 12–17 years [1], and the alarming rates of asthma and its increasing morbidity in urban young people are particularly concerning [2–5]. The disproportionate burden of asthma morbidity in urban children has been attributed to their exposure and heightened sensitivity to certain indoor allergens such as cockroaches or mice [2, 6]. Exposure to some indoor and outdoor allergens has been identified as a major culprit in the development and exacerbation of asthma in pediatric patients [7]. Evidence has consistently shown that children with severe asthma tend to report greater allergic burden [8–11]. Similarly, a large epidemiologic study reported a major impact of allergen exposures and sensitivity on symptom severity and acute healthcare utilization in children with asthma [6].

Despite the wealth of literature elucidating the intricate links between inhalant or food allergen sensitization and asthma morbidity in pediatric populations, the majority of evidence is based on either young children or mixed age groups of young children and adolescents. Little is known about common allergies and their relationships with asthma morbidity specifically among adolescents. Furthermore, a number of studies target only specific allergens (e.g., cockroach or mice), yet there is limited literature comparing various types of allergies and their associations with asthma morbidity. Thus, the purpose of this study was to examine the prevalence of a broad spectrum of indoor/outdoor allergies among urban adolescents and to assess their relationships to asthma control and healthcare utilization.

## Methods

### Settings and sample

Subjects were recruited from US metropolitan cities including Buffalo, New York, Baltimore, Maryland and Memphis, Tennessee. Recruitment strategies included clinician referrals (n = 106), self-referrals responding to school or community outreach (n = 94), study flyers (n = 40), or word of mouth (n = 69). Eligible criteria included (1) age between 12–20; (2) physician-diagnosed asthma that has required healthcare utilization (preventive or acute) within 12 months prior to enrollment; (3) persistent asthma as defined by the National Asthma Education and Prevention Program (NAEPP) guidelines [12]; (4) primary residence located in the participating inner cities based on zip codes or school districts; and (5) ability to understand spoken and written English. Those with other comorbid conditions requiring daily medication (e.g., diabetes, cancer, arthritis, cystic fibrosis, etc.) reported by parents or guardians were excluded.

### Data collection and measurements

The study protocol was reviewed and approved by each Institutional Review Board of the academic institutions in participating cities. Informed consent was obtained from parents and older adolescents (≥ 18 years old), and assent was obtained from adolescents, ages 17 or younger. Data were collected during in-person appointments in the project office, public libraries or in the home. Parents completed a sociodemographic form and forms reporting allergies and current medications. Adolescents ages 18 years or older completed these forms for themselves. Adolescents provided data on asthma control, exacerbation and healthcare utilization.

#### Allergy information sheet

The form assessed nine specific allergies (cat, dog, mouse, ragweed, tree, grass, cockroach, dust mite, and any food) plus other allergies reported by the subjects. Parents were also asked if their adolescent had ever had allergy tests (either skin or blood) or ever received allergy shots.

#### Healthcare utilization and asthma exacerbation

Adolescents were asked whether they had, in the previous 3 months, had asthma related asthma/allergy specialty visits, acute office visits, Emergency Department (ED) visits, or hospital admission. If so, they reported the number of visits and the nights in the hospital. Oral steroid use was also assessed; subjects who used oral steroids for at least 3 consecutive days in the past 12 months were categorized as having asthma exacerbation.

#### Asthma control

Four impairment-based criteria (symptoms, nocturnal awakening, activity limitations and rescue inhaler use in the past 4 weeks) were assessed on a 4-point scale as indicated by the NAEPP guidelines. Based on the criteria, subjects were classified into three categories, well-controlled, not well-controlled, and very poorly controlled. In addition, a total score was computed with higher scores indicating poorer asthma control. We also estimated odds ratios between better or worse control of the teens’ asthma. Instead of using the NAEPP’s three categories of asthma control which resulted in fewer than 15% of subjects having well-controlled asthma, we used the mean to dichotomize the control total score into less than 7.6 (controlled) vs. 7.6 or greater (uncontrolled).

### Data analysis

Healthcare utilization responses were dichotomized into users and non-users of each healthcare service. Logistic regression was fit for each model to predict the probability of utilization and asthma exacerbation with allergy status as a predictor adjusting for subject age and sex. Multiple linear regressions were fit to predict the asthma control (total score) associated with subject allergy status adjusted for age and sex. Estimates of the odds ratio or slope and 95% confidence intervals were calculated along with p-values for the strength of the effect. The Hosmer–Lemeshow goodness-of-fit test was performed to check the predictive power of the logistic regression models. Residual analysis was examined for linear and logistic models to look for outliers, influential points, and overdispersion. No substantial departures from the assumptions were found in these models.

To examine the counts of each type of healthcare utilization outcome, zero-inflated poisson (ZIP) regression was fit for each outcome to predict both the probability of utilization and the change in amount of utilization from allergy status as a predictor while adjusting for subject age and sex. Estimates of the increase in utilization and 95% confidence intervals were calculated and reported with p-values. Residual analysis was examined for each model to look for outliers, influential points, and unusual patterns. No substantial departures from the assumptions were found in these models. To test for differences in allergy effect between those with a sensitivity test (“tested” group) and those without (“never-test” group), we also fit ZIP regression with interactions between allergens and test status. In these models, we estimated the difference between the subjects reporting an allergy in the tested and never-tested groups.

## Results

### Sample characteristics

A total of 313 adolescents from Buffalo NY (n = 123), Baltimore MD (n = 100), and Memphis TN (n = 90) participated in the study. Table 1 summarizes the demographic characteristics of the sample. No significant site differences were found on sociodemographic factors except for the number of non-black participants. The Buffalo subsample had a greater number of white adolescents (32%) than Baltimore and Memphis (5 and 1% respectively) (χ^2^ = 50.3, p < .0001).

**Table 1:** Sociodemographic characteristics of the sample and descriptive statistics of outcome measures (N = 313)

Sex	
Male, n (%)	153 (49)
Female, n (%)	160 (51)
Race	
White, n (%)	45 (14.4)
Nonwhite	
Black or African American, n (%)	246 (78.6)
Multi race, n (%)	19 (6.1)
Others, n (%)	3 (0.9)
Hispanic/latino, n (%)	26 (8.3)
Age, mean (SD)	14.69 (1.96)
12–14, n (%)	149 (47.6)
15–17, n (%)	146 (46.6)
18–20, n (%)	15 (4.8)
Annual household income	
≤ $10,000, n (%)	93 (29.7)
> $10,000 and ≤ $30,000, n (%)	86 (27.5)
> $30,000 and ≤ $70,000, n (%)	80 (25.6)
> $70,000, n (%)	45 (14.4)
Healthcare utilization	
Hospitalization nights, mean (SD), range	0.18 (0.80), 0–7
ED visit, mean (SD), range	0.50 (1.62), 0–20
Acute office visit, mean (SD), range	0.75 (1.38), 0–10
Specialist visit, mean (SD), range	0.67 (1.59), 0–12
Asthma exacerbation, n (%)	83 (26)
Asthma control score, mean (SD), range	7.6 (2.8), 4–16

The majority of the sample (74%) reported an asthma diagnosis before the age of 6. According to the NAEPP criteria, uncontrolled asthma was reported by 85.3% (n = 266), of which 42% (n = 112) were very poorly controlled. The majority of the sample (75%) reported being on at least one controller medication. The most common type of controller medication was inhaled corticosteroids (ICS) followed by the combination of ICS and long acting bronchodilator, 52% and 21% respectively. Almost all participants (97%) reported having a short-acting bronchodilator.

### Prevalence of self-reported allergies

**Fig. 1:**
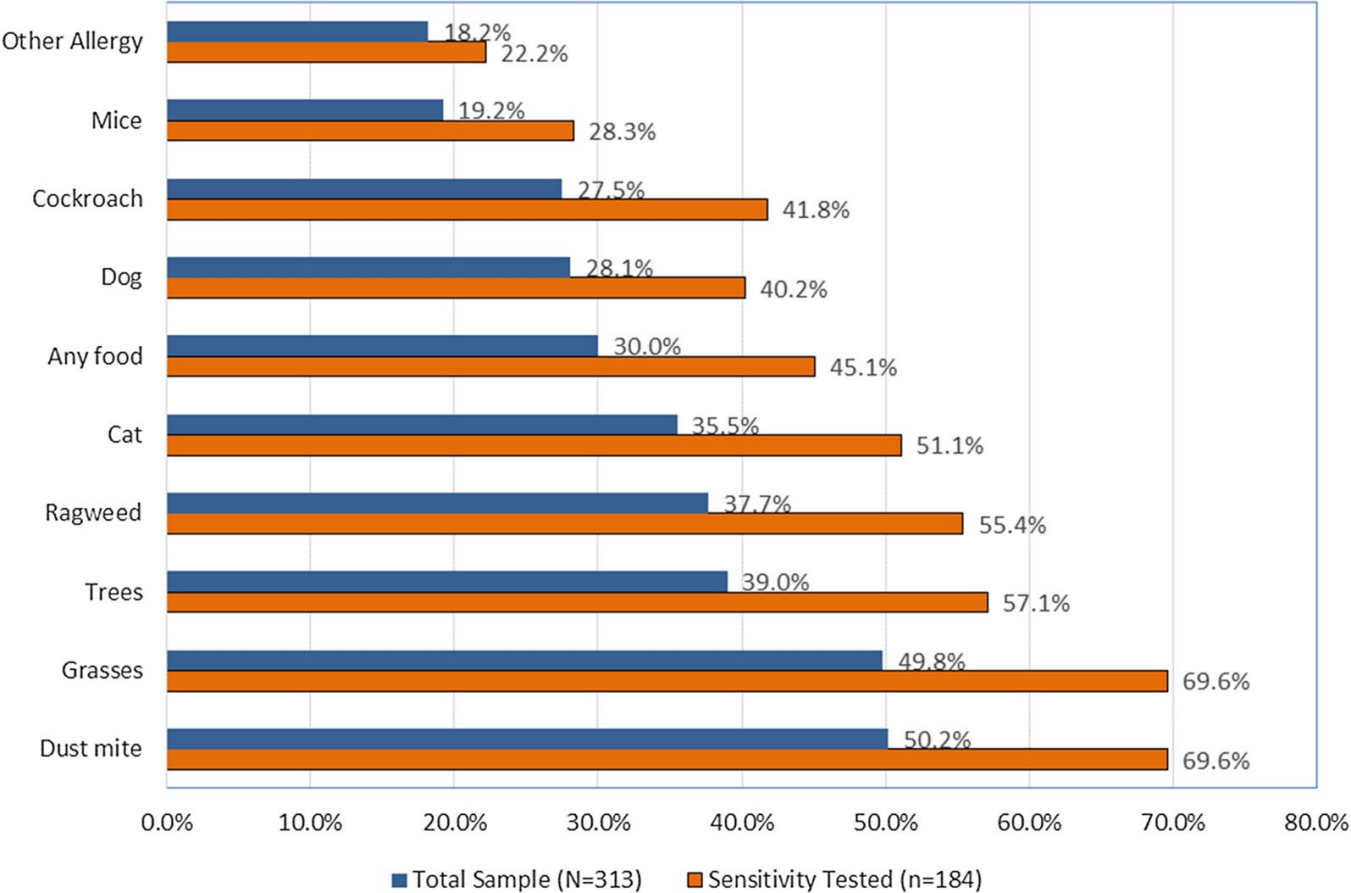
Self-reported common allergies in inner-city adolescents

Dust mites and grasses were the most commonly reported allergies, followed by trees and ragweed (see Fig. 1). Thirty-percent reported some type of food-related allergies. Peanut was the most common food allergen (16%) followed by seafood (10%). At least one type of allergy (maximum of 12 allergies) was reported by 73% (n = 229), of which 87% (n = 200) reported two or more allergies. The average number of allergies per subject was 3.36 (range 0–12). No site or gender differences were found in the types of allergies, except for mice for which Baltimore had significantly higher prevalence than Buffalo or Memphis, 33% vs. 13% or 12%, respectively (χ^2^ = 12.5, p < .001). Despite the extensiveness of self-reported allergies, only 60% had reported having been tested for inhalant and food allergen sensitization, and 15% (n = 46) had ever received immunotherapy (i.e., allergy shots). Figure 1 also shows the prevalence of each of the allergies only in the subsample of adolescents who had ever been tested for sensitivity. Overall, the prevalence of each allergy within the subsample (n = 184) is substantially higher than that of the total sample. Sensitivity to dust mites or grasses was most common, reported by nearly 70% of tested adolescents.

### Allergies predicting healthcare utilization, exacerbation and asthma control

No significant age differences were found on healthcare utilization although older adolescents reported better controlled asthma (0.18 points average lower score per year of age, p = 0.023). Females were more likely to report ED visits (OR = 1.82, p = 0.044), but not significantly different than males in using other healthcare services. Because of the sex and age differences in these outcome measures, these two demographic variables were included in the subsequent prediction models as covariates. Also, cat and dog allergies were grouped into “pets”, and trees, ragweed and grasses into “plants” for subsequent prediction models.

**Fig. 2:**
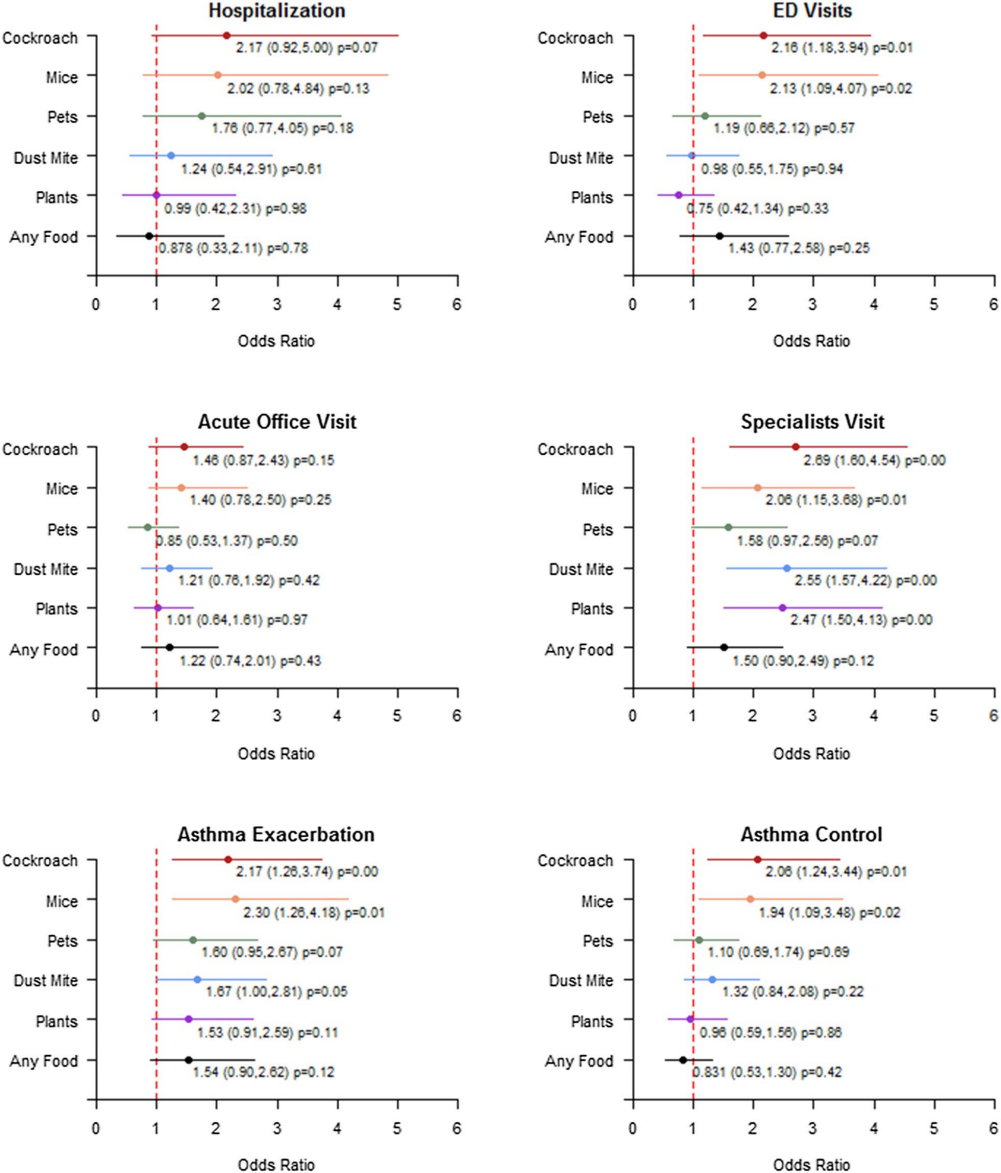
Healthcare utilization, exacerbation, and uncontrolled asthma associated with each type of allergy. Odds ratio (95% CIs) for dichotomous outcome measures after adjusting for age and sex. Odds ratios greater than 1 indicate an increased chance of healthcare utilization, exacerbation, or uncontrolled asthma control predicted by each allergy

Healthcare utilization was significantly predicted by self-report allergies related to cockroaches, mice, dust mites and plants (Fig. 2) after adjusting for age and sex. Both cockroach and mouse allergies were associated with an increase in the probability of ED visits, specialist visits, and asthma exacerbation. Those with cockroach allergy were two times more likely to visit an ED or experience exacerbation, and 2.7 times more likely to use an asthma specialist than those without the allergy. Similarly, the odds of ED visits, specialist visits or exacerbation among those with mouse allergy was 2 to 2.3 times higher than those without the allergy. Dust mite and plant allergies were each separately associated with increased odds of specialist visits by 2.5 times compared to counterparts without these allergies. Pet or food allergies did not predict healthcare utilization, asthma exacerbation or asthma control. Asthma control was predicted by only pest allergies. Based on linear regression models, the control score for subjects with cockroach and mouse allergies were 1.02 (95% CI 0.34–1.70, p = .003) and 0.95 (95% CI 0.18–1.73, p = .017) points higher on average, respectively, indicating poorer asthma control compared to those without such allergies. Figure 2 shows that those with cockroach or mouse allergies are two times more likely to have the total score indicating uncontrolled asthma. Total number of allergies also predicted specialist visits and exacerbation. Each additional number of allergy increased the probability of specialist visits (OR = 1.1, p = 0.001) and asthma exacerbation (OR = 1.1, p = 0.003).

**Fig. 3:**
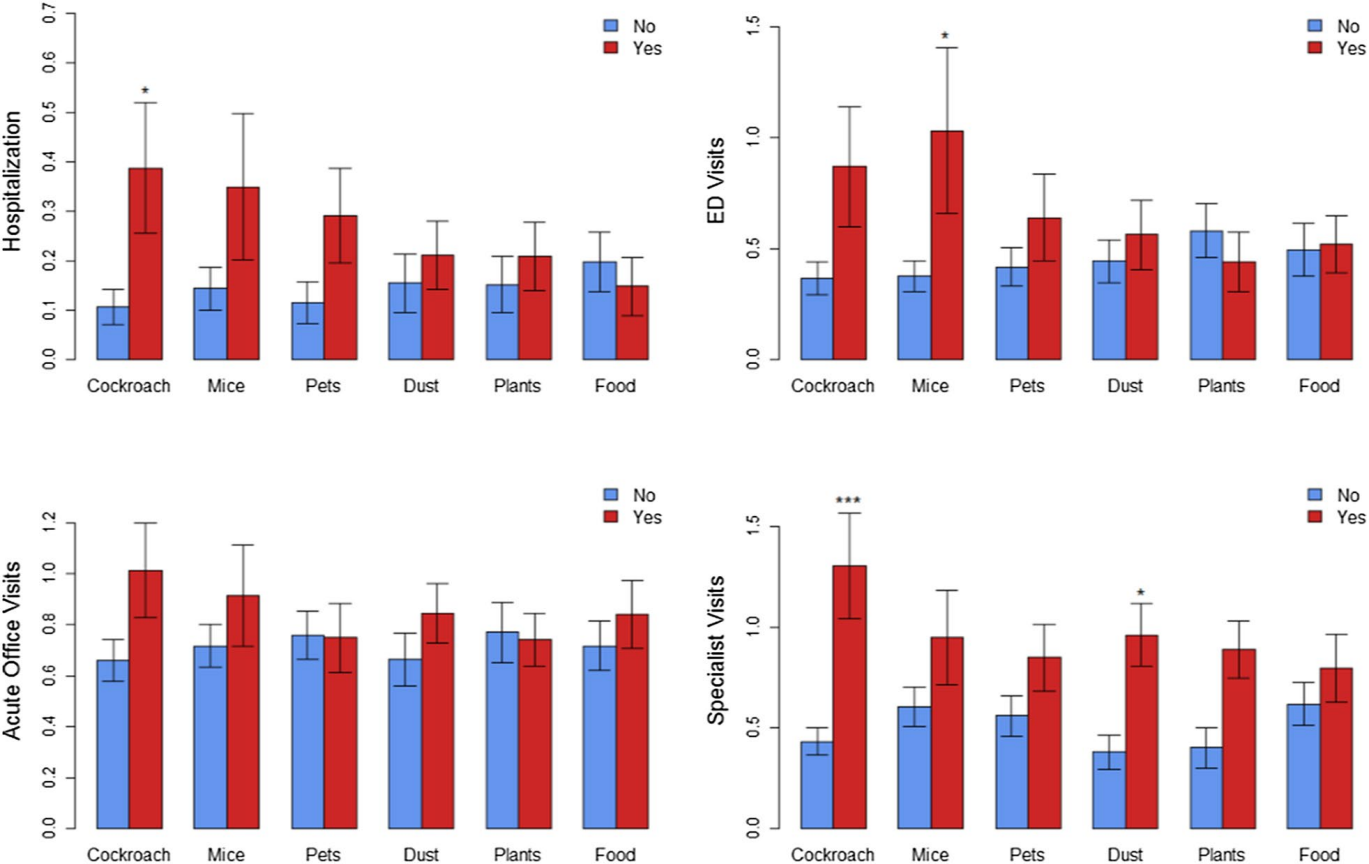
Average number of health care utilizations by each self-reported allergy. The bars represent standard deviations. The asterisks indicate significance of the allergy in the model for counts of utilization from the ZIP model, * for p < 0.05, ** for p < 0.01, and *** for p < 0.001

The average count of each of healthcare utilization for adolescents with and without each of the allergies examined is shown in Fig. 3. The asterisks on each case indicate significant group differences in the number of visits between the groups, after adjusting for age and sex. The change in the number of instances of healthcare utilization predicted by each allergy status was examined in the ZIP models reported in Table 2. Cockroach allergies were associated with 2.2 times as many nights in the hospital (p = 0.036) and 2.2 times as many specialist visits (p < 0.001). Mouse and dust mite allergies were associated with 1.6 times as many ED visits (p = 0.015) and 1.5 times as many specialist visits (p = 0.040), respectively. Total number of allergies also predicted the instances of specialist visits and acute office visits. Each additional allergy was associated with both 1.1 times as many acute office visits (p = 0.012) and specialist visits (p = 0.001).

**Table 2:** Models for the healthcare utilization multiplier predicted by each allergy after adjusting for age and sex (N = 313)

Types of allergies	Hospitalization exp (b)^a^ (95% CI)	ED visits exp (b)^a^ (95% CI)	Acute office visits exp (b)^a^ (95% CI)	Specialist visits exp (b)^a^ (95% CI)
Cockroach	*2.151* (1.053, 3.398) p = 0.0357	1.368 (0.955, 1.960) p = 0.087	1.395 (0.990, 1.964) p = 0.057	*2.152* (1.489, 3.110) p < 0.001
Mice	1.079 (0.536, 2.174) p = 0.832	*1.570* (1.092, 2.257) p = 0.0148	1.076 (0.725, 1.596) p = 0.716	1.029 (0.698, 1.518) p = 0.885
Pets	1.723 (0.805, 3.689) p = 0.161	1.233 (0.852, 1.783) p = 0.267	1.146 (0.811, 1.619) p = 0.441	1.178 (0.837, 1.658) p = 0.348
Dust mite	1.017 (0.532, 1.945) p = 0.959	1.149 (0.785, 1.682) p = 0.474	1.216 (0.856, 1.727) p = 0.275	*1.543* (1.021, 2.333) p = 0.040
Plants	1.687 (0.839, 3.391) p = 0.142	0.876 (0.605, 1.270) p = 0.486	0.976 (0.692, 1.376) p = 0.889	1.210 (0.803, 1.822) p = 0.362
Any food	0.689 (0.307, 1.548) p = 0.363	0.681 (0.455, 1.020) p = 0.063	1.052 (0.735, 1.506) p = 0.782	0.928 (0.647, 1.333) p = 0.687
Number of Allergies	1.031 (0.949, 1.121) p = 0.467	0.984 (0.937, 1.033) p = 0.517	*1.066* (1.014, 1.120) p = 0.0124	*1.090* (1.036, 1.147) p = 0.001

^a^Multiplicative regression coefficients from the zero-inflated Poisson regression: values greater than 1 indicate an increased number of healthcare utilizations predicted for teens with the allergy while values less than 1 indicate a prediction of lower healthcare utilization with the allergy

Sensitivity test status was reported by 309 subjects; 184 have had a sensitivity test (“tested” group) and 125 have not (“never-tested” group). Almost all of the subjects reporting an allergy had been given a sensitivity test (for instance, of 155 teens reporting a dust mite allergy, 128 (82.5%) were in the tested group), making the inference in Table 2 nearly exclusively about the subjects with sensitivity testing. Nonetheless, for each of those models, we checked for differences in the allergy effect on healthcare utilization, depending on their sensitivity test status. In doing so, we used a regression model adding an interaction term between test status (tested vs. untested) and allergy report (yes vs. no) after removing an outlier with 20 recent ED visits. None of the odds of healthcare utilization changed significantly depending on sensitivity test status, but the estimated count of visits was significantly different for five models. For four significant comparisons, the estimated increase in acute office visits and specialist visits was even more pronounced for the “tested” group than the “never-tested” group: expected acute office visits increased for the subjects with dust mite allergy within the “tested” group (337%, p < 0.001), and expected specialist visits also substantially elevated for the subjects with cockroach, mouse, or pet allergy within the “tested” group (376% p = 0.014, 428% p = 0.049, and 1000% p < 0.001, respectively) compared to the “never-tested” group. Only one comparison contradicted the increased healthcare utilization for the subjects with an allergy: among those reporting mouse allergy, there was a 2/3 reduction in the expected number of ED visits for the “tested” group (p = 0.024) compared to the “never-tested” group.

Subsequently, we repeated the ZIP models for each of allergies with only the “tested” group (n = 184) (Table 3). Overall, the effect sizes of sensitivities predicting each type of healthcare utilization were similar to those in the ZIP models with the entire sample (see Table 2) with only two exceptions including cockroach sensitivity predicting hospitalization and mouse sensitivity predicting ED visits for which effect sizes became smaller and statistically nonsignificant. The number of specialist visits remained significantly higher with each additional sensitivity and for those with cockroach sensitivity. Particularly, the number of specialist visits was 50.7% higher in those with dust mite sensitivity, and this effect was significant even after the “never-tested” group (n = 125) were added as shown in Table 2.

**Table 3:** Models for the healthcare utilization multiplier predicted by each sensitivity after adjusting for age and sex only for the subsample with a prior sensitivity test (n = 184)

Types of allergies	Hospitalization exp (b)^a^ (95% CI)	ED visits exp (b)^a^ (95% CI)	Acute office visits exp (b)^a^ (95% CI)	Specialist visits exp (b)^a^ (95% CI)
Cockroach	1.422 (0.322, 6.285) p = 0.642	0.942 (0.517, 1.716) p = 0.845	1.155 (0.746, 1.789) p = 0.518	*2.292* (1.465, 3.585) p < 0.001
Mice	0.470 (0.162, 1.368) p = 0.166	1.036 (0.581, 1.848) p = 0.904	0.730 (0.439, 1.212) p = 0.223	0.798 (0.506, 1.258) p = 0.331
Pets	0.807 (0.167, 3.899) p = 0.789	1.037 (0.584, 1.841) p = 0.902	1.064 (0.672, 1.686) p = 0.792	1.001 (0.673, 1.489) p = 0.996
Dust mite	0.220 (0.032, 1.533) p = 0.126	1.283 (0.587, 2.804) p = 0.532	1.562 (0.853, 2.863) p = 0.149	1.507 (0.885, 2.568) p = 0.131
Plants	1.464 (0.327, 6.552) p = 0.619	0.700 (0.384, 1.277) p = 0.245	0.895 (0.541, 1.481) p = 0.667	1.236 (0.714, 2.139) p = 0.449
Any food	0.912 (0.298, 2.791) p = 0.871	0.785 (0.440, 1.402) p = 0.413	1.142 (0.716, 1.819) p = 0.578	1.037 (0.696, 1.545) p = 0.857
Number of allergies	1.020 (0.868, 1.198) p = 0.811	0.955 (0.883, 1.033) p = 0.247	*1.095* (1.019, 1.178) p = 0.014	*1.092* (1.028, 1.159) p = 0.004

^a^Multiplicative regression coefficients from the zero-inflated Poisson regression: values greater than 1 indicate an increased number of healthcare utilizations predicted for teens with the sensitivity while values less than 1 indicate a prediction of lower healthcare utilization with that sensitivity

## Discussion

This study demonstrates the ubiquitous prevalence of a wide range of self-reported allergies among urban adolescents with asthma, and their associations with asthma morbidity. Dust mites were identified as the most common allergen, reported by more than a half of this population, and the prevalence was strikingly higher, nearly 70%, among adolescents who had been tested for sensitivity. The rate of dust mite allergy in our study is substantially greater than the 35% reported in a previous large study of inner-city children [6]. The difference might be due in part to differences in data sources between our finding and the earlier report, self-report vs. skin testing, respectively. In our study, the self-reported allergy information was based on prior sensitivity testing as well as their experience of allergic reaction to dust mites, thus resulting in a higher rate. Nonetheless, given that the high proportion of adolescents (> 82%) reporting dust mite allergy had been tested for sensitivity, our estimate of mite allergy may closely align with that of sensitivity testing.

We found that allergies to cockroaches and mice were linked to poor asthma control and more frequent ED visits, hospitalization, or specialist visits. Similarly, Busse et al. [6] identified cockroach allergen as a dominant and sole factor of other allergens linked to greater asthma morbidity and urgent healthcare use in young inner-city children. In their study, sensitivity to cockroaches was found in about 37% of the inner-city children; that is slightly higher than our 31% based on self-report. In many urban dwellings, cockroach and mouse infestation is widespread [2, 6, 13], which is often linked to suboptimal living conditions with compromised structural integrity (e.g., cracks and holes in the wall, water leaks) of the house [14, 15]. Cockroach allergens were detected in over 85% of inner-city houses [2], and even when there was no apparent exposure to cockroaches at home, children can become sensitized to the pest from exposures at other places. African American children, 6–16 years of age, are 2.5 times more likely to have cockroach sensitivity than their white counterparts [16]. This alarming rate underscores the importance of a pest-reduction intervention targeting the broader urban community instead of individual houses. Regional differences in the rates of mouse allergy in our study could be due in part to differences in the types of housing. Compared to other sites, Baltimore’s predominant use of multifamily dwellings with shared walls and/or floors/ceilings may provide an ideal environment for the widespread infestation of cockroaches and mice.

The prevalence of self-reported food allergies in our adolescent sample was 30%, a rate slightly higher than that of a previous report in younger children with asthma, 24% [10]. However, our food allergy rate is considerably higher than the prevalence of self-reported food sensitization, 17%, in the general population based on a large U.S. national survey [11]. Similar to the previous reports [11], we found peanut to be the most common food allergen in adolescents with asthma. This is the first study documenting the extent of peanut allergy in adolescents with asthma. In contrast to the previous study [11], in which food allergy was associated with the increased risk of ED visits and asthma exacerbations, we found no significant associations between food allergy and urgent healthcare utilization or exacerbation. This discrepancy may have been in part due to the differences in the sample in the two studies. While the earlier report was based on the general population of all ages, our sample is limited only to urban adolescents. Another reason might have to do with asthma severity. Liu et al. study [11] included those of a wide range of severity, and revealed that food allergy became more prevalent in those with higher levels of asthma severity, thus resulting in ED visits more often. In contrast, all of our participants had persistent asthma and over 85% had not-well controlled or very poorly controlled asthma based the NAEPP classification. The lack of variability due to the relative homogeneity regarding disease severity might have prevented us from detecting any differences in healthcare utilization associated with food allergy. Also, without assessment of specific serum IgE levels, our study is unable to quantify the varying degrees of food sensitivity. If only severe food allergy is linked to asthma morbidity, possible mild cases of food allergy in many of our sample might have contributed to non-significant relationships between the allergy and asthma morbidity in this study.

Plant-based allergies (grasses, trees, and ragweed) were highly prevalent in our urban adolescent sample. Consistent with other studies [17, 18], we found no associations between plant allergies and asthma morbidity, except that those with a plant-based allergy were more likely to receive specialist care for asthma. Pet allergies were also common: however, unlike a previous study demonstrating positive associations between pet allergies and asthma severity [19], we found no relationships between pet allergies and asthma morbidity.

It is noteworthy that the magnitude and directions of associations between certain allergies and healthcare utilization differed by sensitivity testing status. We demonstrated that dust mite allergy was associated with substantially increased urgent office visits due to asthma among the tested group compared to their untested counterparts. Likewise, the positive associations between pest allergies and specialist visits appeared more pronounced among the tested group than their untested counterparts. These findings may simply reflect common clinical practice that sensitivity testing is more often indicated when a patient is presented with severe or uncontrolled asthma necessitating frequent acute office visits or specialist care. When we considered only the subgroup with prior sensitivity testing, however, the relationships between those pest allergies and acute healthcare utilization (ED and hospital admission) became weak and nonsignificant. This may have been due in part to diminished power (sample size) or the possibility that the pest allergies reported by those never tested for sensitivity could be more strongly linked to the acute healthcare utilization than the tested adolescents. The latter speculation is somewhat supported by our finding that the association between mouse allergy and ED visits was negative among those who were tested, indicating fewer ED visits associated with mouse allergy by the tested subjects than those untested. This seemingly counterintuitive finding might have been reflecting the influence of intervening effects, such as treatments (e.g., immunotherapy) or modifications of the home environment to eliminate the pest that were offered to those who tested positive to mouse allergen, resulting in fewer acute asthma episodes requiring emergency care. On the other hand, the untested patients with mouse allergy were left untreated, continuing to suffer greater morbidity represented by more frequent ED visits. Such implication underscores the importance of conducting sensitivity testing for asthma patients reporting pest allergies or living in pest-infested areas to adequately address the issue and ultimately improve asthma outcomes. Future research is warranted to investigate the implications of sensitivity testing for treatment choices and its impact on asthma outcomes in urban adolescents.

This study has several limitations that warrant caution. First, this study relies on self-report data rather than sensitivity testing. Therefore, we cannot rule out the possibility of reporting bias in our prevalence estimation, particularly for about 40% of participants who had never been tested for sensitivity. Those untested adolescents might not be aware of their sensitivity status unless they had prior allergic reactions to certain inhalants or foods, resulting in underreporting allergies. Second, because of the absence of information about specific IgE levels, we were unable to assess how varying degrees of allergic sensitivity play a role in the relationships between certain allergies and asthma morbidity. Third, although this study is based on a relatively large number of urban adolescents representing three discrete regions in the U.S., the sample is by no means representative of urban adolescents in other parts of the country. Moreover, selection bias toward those with higher asthma severity may have occurred as a large proportion (34%) of the sample were referred by clinicians. Future research using a representative sample of urban adolescents is needed to minimize the selection bias. Finally, this study was based on cross-sectional survey data, which limits our ability to making inference about causal links between allergies of various types and asthma morbidity.

Despite the identified limitations, this study offers important insight into the relative prevalence of self-reported allergies of various types in urban adolescents with asthma and the relationships between each type of allergy and asthma morbidity. In general, allergies appear to increase the likelihood of asthma-related healthcare utilization in urban adolescents. Particularly, pest allergies are linked to uncontrolled asthma and exacerbation as well as frequent use of acute healthcare services. Our findings underscore the importance of identifying and eliminating cockroaches and mice that have particular implications for asthma morbidity. Interventions aimed at reducing the level of indoor allergens have been found effective in improving asthma outcomes [20]. Environmental interventions focusing on reduction of cockroaches have resulted in a decrease in the level of the allergens and improved asthma symptoms in urban residents [21, 22]. However, extermination alone may have only minimal or fleeting effects on asthma morbidity unless it is accompanied by patient education and behavior modification that can augment and sustain the effects of environmental interventions. Behavior changes in parents/adolescents to control the pests and to increase treatment adherence in combination with environmental interventions would provide the best chance to achieve enduring optimum asthma control and contain healthcare costs [2].

## Conclusion

This study complements the literature by examining a wide range of self-reported allergies simultaneously, which offered the opportunity not only to compare the prevalence of common allergies but also to assess relative implications of each allergy for asthma burden in urban adolescents. Our findings suggest that not all allergies equally predict increased asthma morbidity. For instance, although dust mite or plant allergies are highly prevalent, their implications for adverse asthma outcomes appear minimal. On the other hand, pest allergies involving cockroaches and mice are consistently linked to greater asthma morbidity. To ameliorate the burden of asthma in urban adolescents effectively, addressing widespread allergies is needed through sustainable interventions modifying urban environments along with increasing patient awareness about allergens and their impact on asthma outcomes. Furthermore, our findings call for clinicians’ careful assessment of allergy status in urban adolescents with asthma, and proactive management of known or potential allergies to prevent and minimize adverse outcomes of asthma.

## Abbreviations

NAEPP: National Asthma Education and Prevention Program
ED: Emergency Department
OR: odds ratio

## References

[1] Center for Disease Control and Prevention. Most recent asthma data. http://www.cdc.gov/asthma/most_recent_data.htm. Accessed 10 June 2017.

[2] Milligan KL, Matsui E, Sharma H (2016). Asthma in Urban children: epidemiology, environmental risk factors, and the public health domain. Curr Allergy Asthma Rep..

[3] Akinbami LJ, Moorman JE, Liu X (2011). Asthma prevalence, health care use, and mortality: United States, 2005–2009. Natl Health Stat Rep.

[4] Akinbami LJ, Moorman JE, Garbe PL, Sondik EJ (2009). Status of childhood asthma in the United States, 1980–2007. Pediatrics.

[5] Akinbami LJ, Moorman JE, Simon AE, Schoendorf KC (2014). Trends in racial disparities for asthma outcomes among children 0–17 years, 2001–2010. J Allergy Clin Immunol..

[6] Busse WW, Mitchell H (2007). Addressing issues of asthma in inner-city children. J Allergy Clin Immunol..

[7] Etzel RA (2003). How environmental exposures influence the development and exacerbation of asthma. Pediatrics.

[8] Genuneit J, Seibold AM, Apfelbacher CJ (2017). Overview of systematic reviews in allergy epidemiology. Allergy.

[9] Hill DA, Grundmeier RW, Ram G, Spergel JM (2016). The epidemiologic characteristics of healthcare provider-diagnosed eczema, asthma, allergic rhinitis, and food allergy in children: a retrospective cohort study. BMC Pediatr..

[10] Friedlander JL, Sheehan WJ, Baxi SN (2013). Food allergy and increased asthma morbidity in a School-based inner-city asthma study. J Allergy Clin Immunol Pract..

[11] Liu AH, Jaramillo R, Sicherer SH (2010). National prevalence and risk factors for food allergy and relationship to asthma: results from the National Health and Nutrition Examination Survey 2005–2006. J Allergy Clin Immunol..

[12] National Heart, Lung, and Blood Institute. Expert panel report 3: Guidelines for the diagnosis and management of asthma. 2007.

[13] Camacho-Rivera M, Kawachi I, Bennett GG, Subramanian SV (2014). Associations of neighborhood concentrated poverty, neighborhood racial/ethnic composition, and indoor allergen exposures: a cross-sectional analysis of los angeles households, 2006-2008. J Urban Health..

[14] Gergen PJ, Togias A (2015). Inner city asthma. Immunol Allergy Clin North Am..

[15] Wilson J, Dixon SL, Breysse P (2010). Housing and allergens: a pooled analysis of nine US studies. Environ Res.

[16] Stevenson LA, Gergen PJ, Hoover DR, Rosenstreich D, Mannino DM, Matte TD (2001). Sociodemographic correlates of indoor allergen sensitivity among United States children. J Allergy Clin Immunol..

[17] Marchetti P, Pesce G, Villani S (2017). Pollen concentrations and prevalence of asthma and allergic rhinitis in Italy: evidence from the GEIRD study. Sci Total Environ.

[18] Palao-Ocharan P, Dominguez-Ortega J, Barranco P, Diaz-Almiron M, Quirce S (2016). Does the profile of sensitization to grass pollen allergens have clinical relevance?. J Investig Allergol Clin Immunol.

[19] Gent JF, Belanger K, Triche EW, Bracken MB, Beckett WS, Leaderer BP (2009). Association of pediatric asthma severity with exposure to common household dust allergens. Environ Res.

[20] Crocker DD, Kinyota S, Dumitru GG (2011). Effectiveness of home-based, multi-trigger, multicomponent interventions with an environmental focus for reducing asthma morbidity: a community guide systematic review. Am J Prev Med.

[21] Morgan WJ, Crain EF, Gruchalla RS (2004). Results of a home-based environmental intervention among urban children with asthma. N Engl J Med.

[22] Gergen PJ, Mortimer KM, Eggleston PA (1999). Results of the national cooperative inner-city asthma study (NCICAS) environmental intervention to reduce cockroach allergen exposure in inner-city homes. J Allergy Clin Immunol..

